# Supporting Citizens in Reflection on Synthetic Biology by Means of Video-Narratives

**DOI:** 10.1177/1075547017730585

**Published:** 2017-09-12

**Authors:** Marjoleine G. van der Meij, Jacqueline E. W. Broerse, Frank Kupper

**Affiliations:** 1 Vrije Universiteit Amsterdam, Amsterdam, Netherlands

**Keywords:** Responsible Research and Innovation, citizen reflection, reflection tools, video-narratives, synthetic biology

## Abstract

To unravel how video-narratives can support reflection in Responsible Research and Innovation contexts, this study evaluates four video-narratives designed for citizen-reflection on synthetic biology. Each video narrative comprised separate clips about three subtopics in which actors represented different views of synthetic biology. The video-clips were presented to Dutch citizens in two different setups: per narrator and per subtopic. Both setups appeared to trigger three reflection processes and three reflection outcomes. Our findings suggest that video-narratives shown per subtopic support reflection more extensively, provided that workshop setups pay considerable attention to reflection on values and assumptions.

## Introduction

Responsible research and innovation (RRI) stands for “the integration and institutionalization of established mechanisms of reflection, anticipation, and inclusive deliberation in and around the processes of research and innovation” (Owen, Macnaghten, & Stilgoe, 2012, p. 755). As this definition emphasizes, RRI calls for processes in which a representative variety of societal actors reflect (Owen et al., 2012; Von Schomberg, 2013) on “purposes and motivations” of research and innovation (R&I; Owen et al., 2012), societal-ethical and technical aspects, as well as “tacit understandings, assumptions, uncertainties, framings and commitments” (Stilgoe, Owen, & Macnaghten, 2013, p. 1575).

During such RRI reflection processes, participants practice and employ their scientific citizenship (Bandelli & Konijn, 2015; Boerwinkel, Swierstra, & Waarlo, 2014; Horst & Michael, 2011). Whereas the learning among participants is often mentioned as a reflection outcome (Broerse & De Cock Buning, 2011), reflection processes on the level of individual participants can be the development and redefinition of the own perspectives (Loeber, Griessler, & Versteeg, 2011), and (re-) consideration of other perspectives, critically or accepting (Davies, McCallie, Simonsson, Lehr, & Duensing, 2009; van der Meij, Kupper, Beers, & Broerse, 2016). On the level of the R&I system, the reflection in RRI contexts can eventually make decision making on R&I more societally responsible and, as a consequence, more inclusive (Korthals, 2011; Stilgoe et al., 2013).

Participants of RRI reflection processes need certain guidance. Emerging R&I fields, which call for upstream, continuous, inclusive reflection processes the most (Sykes & Macnaghten, 2013), are often shrouded in uncertainties (Owen et al., 2012). The methods, approaches, potential applications, and feasibilities are unknown or undecided, making purposes and societal-ethical aspects very difficult to determine (Boerwinkel et al., 2014). Scholars previously identified several process conditions to support the exploration of diversity in RRI reflection, such as the providence of sufficient background information, independent facilitation (Rowe & Frewer, 2000), and the paying of attention to diverse participants (Carpini, Cook, & Jacobs, 2004). The current study aims to look more closely at how tools and formats could facilitate reflection on R&I.

Various studies into informal learning emphasize the power of science narratives and video-narratives about science or technology to stimulate reflection on R&I. Avraamidou and Osborne (2009) identify key components of scientific narratives such as purpose, timing, narrators, and a beginning-middle-end structure. Their usefulness as a tool for reflection lies in the fact that narratives resemble the ways by which the human brain makes sense of the world (Murmann & Avraamidou, 2014). Furthermore, narratives, and especially video-narratives, stimulate a viewer to grow an emotional connection with regard to narrators (Pineda & Bernhardsson, 2011; Walker, Newcomb, & Cagle, 2005). This identification, or reduction of “otherness” (Walker et al., 2005, p. 283), in combination with the rich context that narratives provide such as time, place, and narrator emotions, makes it likely that the viewer respectfully weighs the narrated content (South, Gabbitas, & Merrill, 2008; van der Meij et al., 2016). Moreover, if people view multiple video-narratives about the same topic, they start to assess and compare the narratives, developing understanding of a topic from different perspectives (van der Meij et al., 2016).

The various scientific narrative formats that Avraamidou and Osborne (2009) describe could raise the idea that only scientists are “allowed” to narrate about science and technology to support citizens in reflection on R&I. This aligns with the conventional thinking that a citizen’s science knowledge deficit has to be overcome by communicating scientific knowledge (Bucchi, 2008; Sykes & Macnaghten, 2013). However, in the context of RRI this thinking no longer stands. Namely, in RRI citizens’ framings, assumptions and reflections on impacts are explicitly considered (Owen et al., 2012; Stilgoe et al., 2013; Sykes & Macnaghten, 2013). Therefore, an interesting line of thinking about narratives for RRI contexts is Skydsgaard, Møller Andersen, and King’s (2016) suggestion that reflection is better stimulated if “expert narratives” are combined with “personal narratives,” in which perspectives on R&I derived from lived experiences of nonscientists are shared (p. 48). Such narratives facilitate personal, “deep conceptual learning,” reflection, and discussion on R&I (Skydsgaard et al., 2016, p. 50). In this study, we therefore propose an RRI-specific approach to video-narratives for reflection on R&I, namely, narratives in which semifictional characters realistically and attractively represent citizen viewpoints of R&I in a short and semistructured way.

Various scholars designed, implemented, and evaluated reflection on R&I by means of narratives (Boerwinkel et al., 2014), video (Horst & Michael, 2011), and video-narratives (e.g., Lucivero, 2016; Schmidt, Meyer, & Cserer, 2015) for policy and non–policy-related purposes (Stilgoe, Lock, & Wilsdon, 2014). However, these studies mostly investigate fiction and pay little attention to how video narrative composition contributes to reflection processes and outcomes exactly on the level of the individual participants. Given that R&I reflection processes can have transformative impacts on participants (Davies et al., 2009; Loeber et al., 2011; Sykes & Macnaghten, 2013), further research should consider the relationship between the presentation of video-narratives and their impacts on reflection. This approach would yield insights into the design requirements of video-narratives as supportive tools for reflection in RRI contexts.

This study investigates the presentation of video-narratives, designed to support reflection on synthetic biology (SB), in relation to reflection processes and outcomes. SB is an emerging field in which R&I practitioners use biotechnology to design and build new biological products and systems, with specific preconceived functionalities (cf. Schmidt et al., 2009). “[SB] aspires to move away from genetic engineering guided by trial and error towards a rational design process in which whole genomes can be constructed at the computer” (Boldt, 2016, p. 2). In the past few years, SB has been labelled as controversial (Balmer & Martin, 2008; Torgersen & Schmidt, 2013) since the “discourse on fundamental issues such as biosafety and biosecurity, intellectual property rights, environmental consequences, and ethical and societal implications is still open” (Ancillotti, Rerimassie, Seitz, & Steurer, 2016, p. 309). SB therefore functions as an interesting R&I field for the purpose of this study.

We designed and recorded video-narratives in which four actors, specialized in improvisation, performed a monologue representing citizen narratives of science. The narratives, each portraying a distinct perspective on SB, were constructed based on central assumptions in science and technology studies (Verbeek, 2005) and a series of citizen panels that produced discourses of SB (Betten, Broerse, & Kupper, 2017). Each narrator tells its story in three separate video clips, each covering a particular subtopic, resulting in 12 separate video clips in total. We presented these clips in two different presentation setups to volunteer citizens of diverse age, background, and gender, in both individual and group settings. One part of participants saw all clips of two of the narrators first, followed by all clips of the other two narrators. We hypothesized that this approach would stimulate identification with each narrator, and therefore ensure thorough consideration of the diverse views (cf. South et al., 2008; Walker et al., 2005). The other participants saw video clips per subtopic so that they could, we assumed, compare clips of the four narrators more comprehensively and per subtopic (cf. van der Meij et al., 2016). By analyzing the conversations as a result of the video watching, we aimed to answer the following two research questions:

**Research Question 1:** What reflection processes and outcomes are triggered by the video-narratives?
**Research Question 2:** How do these triggered reflection processes and outcomes differ across presentation setups?

## Reflection on R&I

When people encounter a new situation, like R&I in SB, they tend to engage in a process of inquiry to analyze the situation and make sense of it, with first and second order reflection (Grin & van der Graaf, 1996). First order reflection encompasses the “consideration of problem definitions and evaluation of solutions” (Grin & van der Graaf, 1996, p. 299). At this level of first order notions, effectiveness, risks, problems, costs, and preferred (alternative) solutions are considered (Grin & van der Graaf, 1996; McKee, 2003). For example, at this level people see SB as interesting, promising, potentially dangerous, or unnecessary (e.g., as described in Ancillotti et al., 2016). Second order reflection involves the formulation of reasons behind first order notions, such as values and beliefs (Grin & van der Graaf, 1996). At the level of second order notions, for example, several societal actors in the Netherlands dislike SB, arguing that R&I should not be allowed to freely create all kinds of organisms (Ancillotti et al., 2016). In the elicitation of viewpoints in presence of others, people tend to focus on aspects relevant to their own viewpoint and are often unaware of their second order notions, leaving them unaddressed. Making values and beliefs explicit is, however, a crucial key to dialogue (McKee, 2003). Namely, it facilitates in-depth understanding of one’s own and other people’s perspectives (Drake & Donohue, 1996; Kupper, Krijgsman, Bout, & De Cock Buning, 2007).

**Figure 1.**
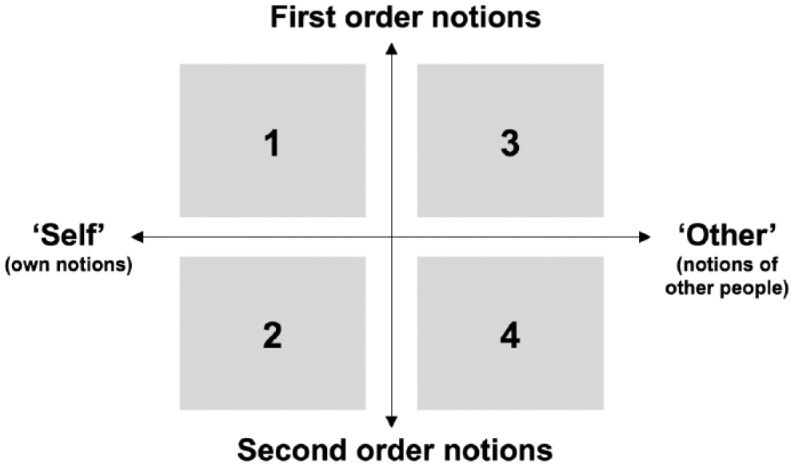
Components of reflection as conceptualized in this study. Note: Quadrant 1 = realization/awareness of own opinion (problems/solutions); Quadrant 2 = realization/awareness of own values and assumptions; Quadrant 3 = realization/awareness of others’ opinions (problems/solutions); Quadrant 4 = realization/awareness of others’ values and assumption.

In this study, we consider that first and second order reflection are crucial for reflection on R&I. Figure 1 shows our conceptualization of the ideal RRI reflection process at the level of a person. Quadrant 1 refers to an individual’s awareness of his or her own first order notions with regard to R&I; Quadrant 2 represents awareness of second order notions. Quadrant 3 refers to awareness of other people’s first order notions, while Quadrant 4 represents a person’s awareness of other people’s second order notions. When reflection covers all four quadrants, a full or complete reflection process is demonstrated. As mentioned above, narratives have the potential to support reflection. We therefore argue that citizen video-narratives of science that demonstrate first and second order reflection might encourage a viewer to reflect on the first and second order notions of himself or herself and of others too.

## Method

### Designing the Prototype

**Figure 2.**
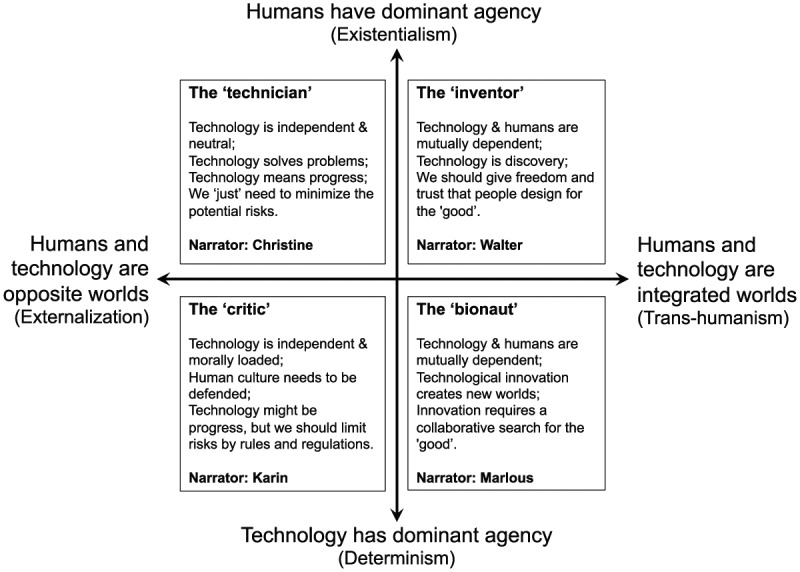
The two axes underlying the four views of synthetic biology about which video-narratives were developed for this study.

Several scholars developed typologies to categorize the ways in which human beings make sense of technology and biotechnology (e.g., Nisbet & Markowitz, 2014; Rogers, 1962; Verbeek, 2005). The technology diffusion model (Rogers, 1962) distinguishes between early adopters, the early and late majority, and laggards. Alternatively, Nisbet and Markowitz (2014) categorize people into groups of science “optimists,” “pessimists,” and the “conflicted,” who see science in a pessimistic as well as an optimistic way (2014, p. 3). Although the latter approach is focused on biotechnology, these typologies give little indication of values and beliefs beyond speed of adoption or attitude. To develop video-narratives for reflection on SB, our challenge was to find a framework that would support both first and second order reflection. Given that the distinction between organisms and technology often determines a person’s view of SB (Müller, 2016), we used a framework that builds on the work of technology philosopher Verbeek (2005) and a study of Betten et al. (2017) into Dutch citizens’ views of SB. The framework positions various assumptions and belief that people can have regarding the relationship between human beings and technology as a basis for sense making related to SB (see Figure 2, derived from Betten et al., 2017).^1^

The left side of the horizontal axis refers to the externalization of technology (Verbeek, 2005): Technology is something separate from human beings. The right side of the horizontal axis resembles Verbeek’s (2005) transhumanist approach, which views technology as fused with human beings. The vertical axis spans the existentialists (top), who approach technology purely instrumentally, versus determinists (bottom). Existentialists see human beings as dominators of technology, and technology as value-free, whereas determinists see technology as value-laden (Verbeek, 2005). In Figure 2, the top-left quadrant demonstrates the mechanic’s view of SB, whereas the top-right represents the inventor. The bottom-left quadrant represents the critic, and we named the bottom-right a bionaut.

**Figure 3.**
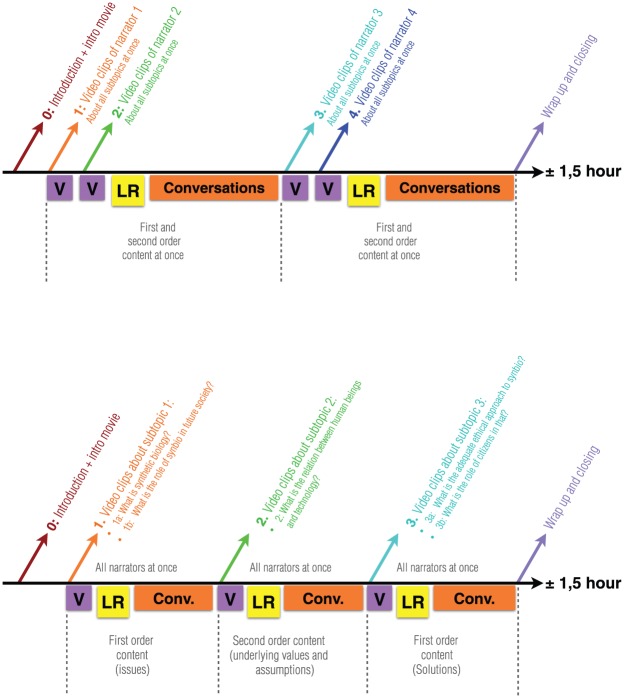
The time lines of the narrator test session setup (top) and subtopic test session setup (bottom). Note: V = video-narrative or video clips; LR = learner report; Conv. = conversation with interviewer/facilitator.

We created narratives for each quadrant of Figure 2 in two steps. First, we aligned their story lines with our conceptualization of reflection on R&I as described earlier, covering first and second order notions in three clips about subtopics that address one or multiple questions:

- Each narrative has an introductory clip about two first order questions, in which narrators problematize SB’s dilemmas and possibilities: (1a) “What is SB to you?” and (1b) “What is the role of SB in our future society?”
- The second set of clips addresses second order notions: values and assumptions underlying the various views of SB (see Figures 2 and 3). These clips cover the following question: (2) “What is the relationship between humans and technology?”
- The final clips address first order notions again, referring to solution finding, by means of the questions (3a) “What is an adequate ethical approach to SB?” and (3b) “What is the role of citizens in the future development of SB?”

After having designed this structure, we recorded the video-narratives by means of scripted reality: We asked improvisation actors to represent one quadrant of Figure 2. Our choice for actors rooted in van der Meij et al.’s (2016) notion that fully authentic narratives, for example, citizens narrating spontaneously in front of a camera, can result in rather lengthy stories that are neither structured nor deep enough for reflection.

During appointments with individual actors, the video director presented Figure 2—the roles of mechanic, critic, innovator, or bionaut, and the subtopics. We asked the actors to invent a name for their character (see at the bottom of each quadrant in Figure 2). After a collaborative identification of several keywords for their narrative, we recorded video clips for each subtopic (see the appendix). Given that narratives are most powerful when they have a clear structure (Murmann & Avraamidou, 2014), the film director and the actors made sure that each clip had a clear beginning and end. From now on, we will refer to the actors as narrators.

### Testing the Prototype

To investigate how the video-narratives evoked reflection on SB, and under which conditions this differed, we organized two group and seven individual test sessions in which a total of nine men and nine women voluntarily took part as participants. Their ages ranged from 17 to 67 years. With the group sessions, we aimed to identify basic reflection processes and outcomes evoked by the presenting of and reflecting on the video-narratives. The individual test sessions were organized to complement the group session data (cf. Lambert & Loiselle, 2008), with the aim to reveal the individual reflection processes and outcomes as triggered by the video-narratives more precisely. The group test sessions took place at Wetlab evenings (http://waag.org/en/lab/open-wetlab) in Amsterdam with two groups of seven and four adult participants, respectively. These participants were visiting an *Introduction to Do-It-Yourself Synthetic Biology* event, so they were novices but they were probably more interested in SB than average citizens. The individual test sessions took place in Amsterdam, Utrecht, and Nijmegen, with citizens who were unacquainted with the field. In these sessions we aimed for a spread in gender and in knowledge of R&I. The individual participants were indirect acquaintances of the researchers and research assistant. Participants of the group test sessions did not participate in individual test sessions, and vice versa.

We presented video-narratives in a narrator presentation setup in one group session and four individual test sessions, and a subtopic presentation setup in the other sessions (see Tables 1 and 2 and Figure 3). In the latter, we presented narratives of all four narrators subtopic by subtopic, each immediately followed by reflective conversations, aiming to trigger intervideo comparison on individual subtopics. This resulted in sessions with three viewing and conversation rounds, namely, one round for each subtopic. In the narrator setup, we presented full video-narratives of two narrators, aiming to trigger closer identification with each narrator. As a result, these sessions had two rounds, namely, the viewing of and conversing about videos of two narrators at a time. We varied the sequence in which the narrators were shown. Both setups took 1½ hours and comprised the following steps:

1. We gave an explanation about the study, emphasizing the neutrality of the facilitator to prevent bias; all different viewpoints were welcomed.
2. We asked informed consent for audio recording for anonymous data analysis.
3. The presenting of a short video introducing SB, plus conversing with individual participants on their initial view of the field. We derived this video from a presentation used in Betten et al. (2017). The video presented technical principles of SB and its promised possibilities in various application domains, mentioning that there are currently unknown uncertainties and risks too.
4. Several rounds of watching video-narratives (two rounds in the narrator setup, three rounds in the subtopic setup, see Figure 3).
5. After each round of video watching, participants were asked to fill in a Learner Report (LR; Van Kesteren, 1993), which comprised six sentences without an end, requiring participants to complete these sentences. As LRs are meant for individual reflection on learning (Van Kesteren, 1993), we designed the reports to make participants reflect on their responses to the videos, and relate the views of the narrators to their own view (“After seeing this video, I became aware of/was surprised/(un)pleased that/know that I/others . . .”).
6. We held active conversations with the participant(s) about the videos: “What do you think about Christine/Karin/Walter/Marlous?” (in the narrator setup) or “What do you think about these viewpoints?” (in the subtopic setup). The interviewer limited its role to one “deepening” question per round (e.g., “How come you see it this way?”), to gain insights into the extent to which first and second order reflection would spontaneously happen after participants had seen the videos.
7. An end-reflection, in which we conversed with the participant(s) about what had happened during the test session.

**Table 1.** Group Test Session Participant Characteristics and Session Setup Details.

Group session	Participant	Gender	Age (years)	Education	Test session setup details
Session 1	GP1	Male	23	Higher vocational education	N: CKWM
	GP2	Male	25	University MSc	N: CKWM
	GP3	Female	25	Higher vocational education	N: CKWM
	GP4	Female	25	University MSc	N: CKWM
	GP5	Female	25	Higher vocational education	N: CKWM
	GP6	Female	27	University MSc	N: CKWM
	GP7	Female	31	Higher vocational education	N: CKWM
Session 2	GP8	Male	25	University MSc	ST: KMCW
	GP9	Male	25	Higher vocational education	ST: KMCW
	GP10	Male	26	University MSc	ST: KMCW
	GP11	Female	28	University MSc	ST: KMCW

Note: ST = subtopic setup; N = narrator setup; C = Christine; K = Karin; W = Walter; M = Marlous.

**Table 2.** Individual Test Session Participant Characteristics and Session Setup Details.

Individual session participant	Gender	Age (years)	Education	Test session setup details
IP1	Male	27	University MSc	N: WMCK
IP2	Female	27	PhD	ST: CKWM
IP3	Female	24	Higher vocational education	ST: WCMK
IP4	Male	36	Higher vocational education	N: WMKC
IP5	Female	65	University MA	N: KMCW
IP6	Male	67	Higher vocational education	ST: KWCM
IP7	Male	17	High school HAVO	N: WKCM

Note: ST = subtopic setup; N = narrator setup; C = Christine; K = Karin; W = Walter; M = Marlous.

#### Data Gathering, Reduction, and Processing

The test sessions were anonymously transcribed verbatim, including the interviewer’s texts. For the labeling, we chose to reduce the data to the level of sentences (DeCuir-Gunby, Marshall, & McCulloch, 2011). To make analysis of reflection possible at the level of individual participants, we separated group session transcripts in sentences per participant. However, for meaningful analysis we needed to maintain certain contextual information surrounding the sentences of each individual participant. In the individualized transcripts, we therefore included facilitator questions and some other immediately preceding contributions of other participants too. Last, we also inserted the sentences that participants wrote in their LRs in the conversation transcripts at moments in the session that participants had done the LRs.

#### Data Analysis and Coding Strategy

We coded the transcripts, including the LR answers, by using a combination of deductive and inductive coding (Braun & Clarke, 2006), comprising iteration between the revision of theory and repeated examination of raw data (DeCuir-Gunby et al., 2011). First, the lead author coded transcripts on the occurrence of first and second order reflection as conceptualized above (Grin & van der Graaf, 1996). Second, based on the initial coding, the first author identified several data-driven themes, with particular attention to identifying reflection processes and outcomes. Examples and nonexamples of themes were discussed and refined with the third author to determine the reliability of the codes and the coder (cf. DeCuir-Gunby et al., 2011). Differences in opinions between researchers were resolved by discussion among the authors. This led to the identification of several themes in the transcripts.

Third, we compared these identified themes to the perceptions of participants on what had happened during the session, as expressed in their LRs or during conversations with the interviewer. These perceptions were used to verify the themes as identified by the authors and inductively yielded three reflection processes and three reflection outcomes.

## Findings

We identified three reflection outcomes and three reflection processes that were dominantly triggered by reflection on the video-narratives. The various processes and outcomes were demonstrated simultaneously during conversations: The reflection outcomes and processes did not exclude one another and even reinforced one another. Table 3 shows how often each reflection process and outcome occurred in the different setups. As 11 citizens participated in the narrator setup and 7 in the subtopic setup, we also calculated the “relative instances”: We divided instances of reflection processes and outcomes in narrator sessions by 11, and instances that occurred in subtopic sessions by 7 (see Table 3).

**Table 3.** Overview of Reflection Process and Outcome Instances, Distinguishing Between Conversations (Conv.) and Learner Reports (LRs), Narrator (N) and Subtopic (ST) Setup, the Total Number of Instances, and Instances Corrected by the Number of Participants Per Setup (in Parentheses).

	Conv. N setup (*n* = 11)	Conv. ST setup (*n* = 7)	Total in all conv. (*n* = 18)	LRs N setup (*n* = 11)	LRs ST setup (*n* = 7)	Total in all LRs (*n* =18)
Reflection outcomes						
Minor modification	11 (1.00)	4 (0.57)	15 (0.83)	4 (0.36)	0 (0.00)	4 (0.22)
Analytical understanding	10 (0.91)	6 (0.86)	16 (0.89)	4 (0.36)	5 (0.71)	9 (0.50)
Diversity appreciation	7 (0.64)	4 (0.57)	11 (0.61)	10 (0.90)	8 (1.14)	18 (1.00)
Reflection processes						
Shopping (first order)	23 (2.09)	18 (2.57)	41 (2.28)	18 (1.64)	17 (2.43)	35 (1.94)
Demarcation (first order)	21 (1.91)	17 (2.43)	38 (2.11)	24 (2.18)	23 (3.29)	42 (2.33)
Understanding fundamental differences (second order)	2 (0.18)	8 (1.14)	10 (0.56)	3 (0.27)	4 (0.57)	7 (0.39)

### Reflection Outcomes

Comparing participants’ final and initial discourses at the beginning and end of test sessions, respectively, we identified three common reflection outcomes: (1) participants slightly modified their initial views by hearing and reflecting on the viewpoints represented in the video-narratives; (2) participants developed more analytical understanding of narrators’ perspectives, even when they were totally different from their own; and (3) participants started to value the existence of a multiplicity of perspectives as represented in the video-narratives. The following quote of group session participant GP11 summarizes these findings:

(. . .) I’ve not really changed my opinion but it might lead me to be more wary of dismissing other people’s viewpoints.

We provide details of each outcome in further detail below. Quotes were translated from Dutch and adjusted for readability purposes.

#### Minor Modifications

During test sessions, video-narratives increased participants’ vocabulary and knowledge of SB, but their general tone of language about the field remained. When participants’ initial view of SB was enthusiastic or hesitant, they continued to hold this view until the end of the session. We traced minor changes in views during conversation (15 instances among 10 participants). Four participants reported in LRs and reflective conversations that they had slightly changed their views, illustrated by the following quote of individual test session participant IP4:

In the beginning, I only thought of risks. (. . .). But later on I saw more possibilities too. (. . .) As you are always influenced. (. . .). The more things are being told, the more it starts to work in your mind; it starts to live.

#### Analytical Understanding

Throughout the process of watching video-narratives and conversing about the content, participants developed an enlarged analytical understanding of other views (16 instances among nine participants). At the beginning of test sessions, participants could be critical and sometimes even become slightly annoyed by narrators with views different to their own. As the sessions progressed, participants started expressing certain understanding of these different views. Test session participants who initially identified themselves with the opinions expressed by Karin (critic) seemed to gain understanding later in the session for the viewpoints of Christine (mechanic) or Walter (inventor). Alternatively, participants who observably resembled and identified the most with Christine, Walter, or Marlous seemed to develop certain understanding for Karin’s arguments too. The following quote of individual test session participant IP1 illustrates the growth of analytical understanding. Throughout the session, the participant had repetitively expressed disagreement with Karin’s critical stance toward SB. However, almost at the end of the session, the participant said the following:

Karin does hit a nerve there by [noting that] (. . .) if everybody could decide the gender of their child in the future (. . .), the consequences could be enormous. Although I don’t agree with her way of looking at it, she makes you realize that this is how far it could go.

After this, the participant began to consider the need for thinking about regulations on SB, like Karin, although not agreeing with Karin’s views in many respects. To summarize this identified reflection outcome, the viewing and considering of video-narratives seemed to increase participants’ understanding of other views of SB.

#### Appreciation of Diversity

Often coupled, but representing a more generic outcome than increased analytical understanding, participants started to value the existence of a multitude of views of SB as represented in the video-narratives (11 instances among 10 participants). For example, group session participant GP10 wrote, after seeing video-narratives about “What is SB?” and “What is its potential impact on our future society?” in the LR:

Perhaps it could be said that the first and last viewpoints represent ‘society’ [Karin], ‘humanism’ [Walter], while the other viewpoints are focused on practicalities, process, technology [Christine & Marlous]. All viewpoints are needed.

Several times in conversations during this session, this participant again shared her appreciation of diverse perspectives. It seemed that the video-narratives with various perspectives on the topic helped participants to discover a value in multiple perspectives. Given that the LRs showed 18 additional instances of this reflection outcome (see Table 3), it could be the case that this increased appreciation of diversity occurred more often than expressed in conversations.

### Reflection Processes

Looking at the ways in which participants reflected on the views represented in the video-narratives and their own view, respectively, we identified three dominant processes. We identified two processes of reflection at the level of first order notions: shopping and demarcation. One less common process had a deepening character in which second order reflection also took place.

#### Shopping

The video-narratives had the effect of encouraging participants to combine and jump back and forth between the four narratives to articulate their own view (41 instances among 17 participants). The conversation fragments below, from individual test session participant IP7, illustrate this shopping process. After seeing the introductory video, the participant cautiously shared an initial view of SB:

Well, it seems good in itself (. . .). I saw that people are already creating new limbs (. . .). Things like that would be really useful. (. . .). [I] think that they’re going to do it especially in the medical field or something. (. . .) But I don’t know, maybe they can do bad things with it. Not that I know what. (. . .) If [this technology] gets into the wrong hands (. . .) Yes, that might just happen. Of course I don’t know exactly what people can do but if it did that would not be good.

Subsequently, after seeing the full video-narratives of Walter and Karin, the participant commented,

Yes, I would say we should not mess about with nature too much. It would make the ecosystem fall apart. But do I think we can give nature a little push, steering it in the right direction.

In this quote, IP7 emphasized the cautious arguments of narrator Karin combined with the optimistic arguments of narrator Walter. Incorporating the views of these narrators helped this participant enrich the initial pro and contra arguments as cautiously opted before viewing the videos. IP7 reflected on the following during the process:

Well it [seeing Karin and Walter] helped me a bit to form an opinion because they show a little bit about two sides.

Another participant of an individual test session confirmed this process as well, while reflecting on the session at the end:

(. . .) These people show you angles that you would not have thought about otherwise. So that’s the nice thing about it. It helps you to form your opinion.

The 41 instances of shopping show that this process made participants develop a more precise view.

#### Demarcation

Participants of our test sessions also defined their own view by highlighting aspects of video-narratives with which they disagreed (38 instances among 15 participants). This reflection process generally occurred when participants were less hesitant to develop their own view, and when they were confronted with narratives that they perceived as different from their own. An illustration of this demarcation comes from a group session. After the facilitator asked participants to respond to the content of the introductory video, participant GP6 noted:

(. . .) I’m worried about the governance of the innovations in synbio. (. . .) I was wondering how funding is applied and how that steers in some way. What type of research is going to be done and how is it going to be applied?

These immediate concerns slightly resemble the narrator Karin in terms of having concerns but also the narrator Christine in terms of minimizing the risks of innovation. However, after having seen two full video-narratives of Christine and Karin, the GP6 mentioned in the LR:

After seeing the film clips, I know that I feel I am caught between both clips. I can understand the rhetoric of both [Karin & Christine], and yet I do not agree fully with them.

In the LR, GP6 also explained her disagreement with Karin:

I was surprised by that conversation on the relationship between humans and technology in Clip 2. [Karin] places technology as an ‘other,’ something outside ourselves, when in fact it is part of us.

During the plenary conversation, the participant commented about her further disagreement with the video characters:

Christine mentioned that the debate should not be led by fear. But is fear not just always present in making decisions for choosing how to implement synbio? For instance, if people say, “For health reasons it’s okay, but for other reasons it’s not okay,” isn’t that also driven by fear? The fear of death. Is that a rational decision? You can’t really say it’s a rational debate. It is never a rational debate.

This inquiry into the video-narratives that were initially comparable with the participant’s own initial view created further boundaries on the participant’s views.

#### Discovering Fundamental Differences and Similarities

Although the above quotes show certain second order discourse, deeper reflection on, for example, why one should have concerns regarding SB or why is it important to be optimistic about SB, was generally absent in most of the shopping or demarcation instances. We could identify only 21 instances of second order reflection in conversations (and 7 additional instances in LRs) that revealed analysis of values and assumptions underlying their own or other views of SB. Ten of these 21 instances could be clustered as a pattern (see Table 3), namely, that participants discovered why they agreed or disagreed with particular narrators. The quote of individual test session IP2 illustrates this:

Well, I agree the most with what Christine said. I think this is also why I felt so hesitant in my reaction to Karin in the previous video; she really pretended as if the development stands by itself and that [humans] have no control over it. [Christine] articulated that it is ultimately humans who decide. Developments may come unexpectedly but ultimately it is humanity who makes the reasonable choice about what happens with it. And I agree the most with that. And that whole story of Walter about that film he refers to in which humans and technology coalesce, really sounds like a nasty nightmare. [Laughing] (. . .) He may think this but technology is a sort of utensil and thus we ultimately decide what happens. Something that makes life easier and makes it better, and not something that sort of merges with us . . . like in that film.

IP2 discovered that his own position was closer to Christine’s because he disagreed with the underlying assumptions of Karin and Walter.

### Influences of the Setups, Prototype, and Facilitation Techniques

Although the test scale was small, several differences could be observed between the narrator and subtopic setups. Overall, if corrected by the number of participants per session (see Table 3), the subtopic setup especially triggered reflection processes more powerfully than the narrator setup. First, in the subtopic setup, shopping occurred 18 times versus 23 times in the narrator setup. Correcting these instances by the number of participants (7 vs. 11), the subtopic setup triggered shopping more extensively. Also, 35 instances of shopping could be identified in the LRs, indicating that the LRs might have been a major trigger of this reflection process. Second, some 17 of the demarcation instances occurred in the subtopic setup versus 21 instances in the narrator setup. Correcting these instances by number of participants (see Table 3) indicates that the subtopic setup triggered demarcation more extensively. We noted that LRs showed 42 additional instances of demarcation. Therefore, we postulate that participants may have been more hesitant to express disagreement with opposing viewpoints in the face-to-face conversations, especially in group sessions, in which individual participants had less time to talk. Third, the subtopic setup seemed to trigger second order reflection more extensively than the narrator setup (8/10 instances), particularly the video clips about “What is the relation between humans and technology?” These setup differences suggest that the sub-topic setup carries more potential for thorough first and second order reflection on R&I compared to the narrator setup.

We also noted several setup overarching effects. First, the introductory video triggered the initial reflection process. It was designed to be neutral, namely, explanatory about the technologies used in SB and indicative with regard to possibilities and risks, without promoting or overcriticizing the field in content or tone of voice. The content was derived from an SB introduction presentation as tested in Betten et al. (2017). Still, participant GP10 mentioned in response to the introductory video:

I thought there was some surprising imagery used. In particular, the DNA feeding into this kind of like iconic factory with DNA coming out of the other side (. . .) I think a lot of people are concerned about the industrialization of human life. So Uhm, yeah to me that [video] kind of triggered this thought.

Indeed the introductory made use of several schematic illustrations, which could have framed SB in particular ways. However, participants did not articulate extreme resistance or extreme enthusiasm toward SB after the introductory video with references to its visualizations. Most participants moderately emphasized potentially positive and negative aspects of SB in response to the introductory video, although at that time they could not always articulate their precise views.

Second, we noted eight instances in which participants were annoyed by particular characters as the following quote of a group session participant illustrates:

(. . .) The DJ reminds me of like people of my generation that just know little bit about the technology and are, like, ‘yaaay technology!’

However, just as frequently, participants were inspired by particular narrators. This annoyance and inspiration was particularly generated by Walter, the innovator, who had many ideas about what people should be able to do with SB.

Third, we ensured that each video clip lasted less than 3 minutes. However, our participants could not always remember the narratives; three participants mentioned this explicitly during conversations. This memory issue might have had an impact on the depth of participants’ reflections with regard to the content of the video-narratives.

Fourth, explicit deepening questions from the facilitator (e.g., “How come you see it in this way?”) seemed to trigger more extensive, deeper reflections. Most discoveries of fundamental differences arose after explicit questioning of the facilitator. Last, the participants’ answers to the unfinished sentences in the LRs, such as “After seeing this video narrative I know that . . .” and “I was surprised/pleased/unpleased that . . .,” seemed to encourage participants to analyze each of the narratives during conversations in more depth—“I agree with (name), but I don’t agree with (name).” In particular, the unfinished sentences “I liked . . .” and “I didn’t like . . .” appeared to trigger participants to engage in shopping and demarcation.

## Conclusion and Discussion

In this exploratory study, we found that reflection on video-narratives of four different views of SB induced several valuable reflection processes and outcomes. The video-narratives facilitated development of understanding of other views among our test session participants. As a result of the video-narratives, participants appeared to appreciate the diversity of perspectives on SB without completely abandoning or disrupting their own initial views. These reflection outcomes emerged from a second order and two first order reflection processes. The latter concerned shopping, in which participants carefully collected aspects of various videos to construct and express their own view, as well as demarcation in which participants defined what they thought of SB by disagreeing with particular aspects of the videos. On the rare instances of second order reflection, participants became more aware of their reasons for agreeing or disagreeing with particular aspects of the video-narratives.

Our findings demonstrate differences between the impacts of the two setups in which we tested the video-narratives. A setup in which videos were presented on the basis of subtopics resulted in relatively more instances of reflection processes and outcomes, when corrected for the greater number of participants. Moreover, almost all instances of second order reflection occurred in the subtopic setup, particularly after viewing video-narratives on the subtopic “What is the relationship between human beings and technology?” In addition, the facilitator and LRs played an important role in triggering reflection outcomes and processes.

These findings suggest that the presentation of video-narratives can be useful for inclusive reflection in RRI contexts. We have identified not only several ideas on how to present video-narratives for the stimulation of different reflection processes and outcomes but also several challenges in making the video-narratives recognizable, immersive, and contextually qualitative to actually achieve the occurrence of these reflection processes and outcomes. In the next section, we therefore share insights into the design of video-narratives for reflection on R&I. After this, we reflect on the value of video narrative–based reflection in RRI contexts.

### Design Implications

First, our findings indicate that video narrative–based reflection does require complementary tools and exercises to deepen the reflection. Our findings showed that video-narratives did not trigger much second order reflection on their own. LR questions (cf. Van Kesteren, 1993) and facilitation were useful additional means to deepen reflection. From this, we consider that strong facilitation and additional tools, such as PlayDecide cards (Bandelli & Konijn, 2011), could be incorporated in video narrative–based reflection to guide participants into deeper reflection.

Second, previous research on video-based learning has shown that videos benefit from a strong topical focus to guide the learning and reflection (van der Meij et al., 2016). Our findings confirm this notion. The subtopic setup seemed to facilitate second order reflection (Grin & van der Graaf, 1996) slightly better than the narrator setup. In the subtopic setup, second order discourse could be seen directly after showing videos on the question “What is the relationship between human beings and technology?” As a result, we argue that video narrative–based reflection processes need to be divided into small steps of reflection. They should include a separate moment for the viewing of video-narratives about second order notions concerning the R&I at stake, followed by reflection.

Last, video narrative–based reflection requires videos with narrators with seemingly equal knowledgeability, importance, seriousness, and humor. One of our narrators, Walter, appeared to annoy our test session participants more than the other characters. Although this can be a matter of taste, this narrator had a more indifferent, informal attitude when compared to the other narrators, possibly triggering participants’ tendency to abandon his viewpoints. Murmann and Avraamidou (2014) noted that the quality of actors strongly determines the impact of narrative-based science learning. Although videos provide rich context that promotes immersion and facilitates learning (Pineda & Bernhardsson, 2011; Walker et al., 2005), we argue that Walter’s acting style and role might have negatively overwhelmed our participants. If this was the case, annoyance inhibited the degree to which his views could be seriously assessed. Therefore, it might be useful to make actors collaborate with one another in making video-narratives, to equalize their vocabulary, vividness, and talking speed and even the duration of their stories.

### The Added Value of Video-Narratives for Reflection on SB

Reflection on first and second order notions is important if dialogues are to result in mutual understanding (McKee, 2003). In reflection without additional tools, the facilitator has to assure that attention is paid to diversity in order to encourage participants’ understanding for diverse views (Rowe & Frewer, 2005). It is a limitation of this study that no experimental design was applied with a control group that reflected on SB without videos or with unrelated videos. We made this choice, because we were looking for a qualitative in-depth understanding of the ways by which our participants made sense of the different citizen narratives, rather than examining effect relationships between the stimulus and group discussions. Nevertheless, a possible comparator is the study of Betten et al. (2017), which found that citizens can voice issues and concerns regarding SB when put together in a citizen panel but reflect less explicitly on the underlying structure of those issues and concerns by default. This, we argue, is the added value of video-based narratives. Namely, our video-narratives, showing four different perspectives on SB, supported participants to dive into the rationales of various viewpoints and thereby develop analytical understanding and appreciation for diverse perspectives.

Additionally, we used a new format for science narratives. We did not present an action story that shows a major problem to which SB delivers a solution, neither did we present a movie in which a “visually attractive researcher” excitingly explains the promises and risks of SB. In contrast, we made improvisation actors narrate monologues to represent citizen views of SB as identified in Betten et al. (2017). We had structured the narrative rationales by means of Verbeek (2005) and composed the monologues by making them cover a problematization of SB, values and assumptions underlying these, and then the ethical approach to SB (cf. Grin & van der Graaf, 1996). These narrated monologues do not fit in the narrative categorization of Avraamidou and Osborne (2009). Based on this experience, we argue that our new format for narratives is interesting for the facilitation of reflection on SB, in addition to more common forms of narratives such as science fiction (Knippels, Severiens, & Klop, 2009) or artist impressions of SB futures (Schmidt et al., 2015).

Dewulf and Bouwen (2012) identified various strategies of interaction between people’s views in face-to-face conversations: incorporation, disconnection, polarization, accommodation, and reconnection. With regard to reconnection they argued, “Reconnecting faces the double challenge of taking one’s own and the challenging issue framing simultaneously as serious and finding a workable relation between them” (p. 188). We perceive a link between Dewulf and Bouwen’s favorable application of reconnection and the shopping process that we identified in our study. After seeing the video-narratives, our participants seemed to identify overarching patterns in two or more narratives in order to further develop their own view, and they also occasionally embedded these overarching elements into their view. A larger study may be needed to identify whether this process resembles Dewulf and Bouwen’s frame reconnection. We consciously designed the video-narratives to show context and rationales underlying different views, which may have made them all plausible enough for serious consideration of their content seriously. Consciously composed video-narratives, covering both first and second order notions, may support the growth of respect for diverse perspectives. Assuming that this leads to mutually respectful conversations, video-narratives could be of great value for dialogues that aim to stimulate mutual learning.

### The Role of Video-Narrative–Based Reflection for RRI

Deliberative reflection processes constitute a crucial element of the responsible embedding of science and technology in society (Stilgoe et al., 2013). Such deliberative processes are challenging to organize because the diversity of views and backgrounds can lead to polarization, inhibiting fruitful reflection on values, purposes, goals, and futures of science and technology (Abelson et al., 2003; Carpini et al., 2004; Rowe & Frewer, 2005). We argue that reflection exercises supported by semiscripted video-narratives may contribute to fruitful deliberation. Video narrative-based reflection seems to lower the threshold for people to interact and say what they think, creating the opportunity for diverse views to be expressed and heard. Such respect for diversity will become increasingly crucial if commonly agreed pathways and future visions of science and technology are to be developed.

## Appendix

**Appendix:** Transcripts of Video Narratives.

	Christine	Karin	Walter	Marlous
Subtopic 1a: What is synthetic biology?	For me it’s about being able to create your own world. I’m not a biologist so I’m not familiar with the real subject but this is how I see it. And I think with synthetic biology we will have a toolbox available, with all kinds of solutions for problems we have right now.	To be honest I didn’t know what it was until you told me a little bit about it. Of course I have read articles and stuff. In my opinion it’s a kind of . . . artificial biology, human made biology, and I think we should be cautious. We should be careful with this.	I think it’s when you play with biology. So it’s like creating new life, by using technology. It’s something everybody can do. Like I make music with computers, so you can make new life with technology.	(In Dutch) Synthetic biology as far as I can see, is actually a new development. I see it as a continuation of scientific developments in the field of genetic technology or nanotechnology. It will develop a sort of new order (of life).
Subtopic 1b: What is the role of synthetic biology in our future society?	I think it will help us to be way more efficient in solving problems; big problems that we have right now. Like we have overpopulation of the earth hanging above our head. And this is going to help us to produce food more efficiently, I think. Another thing is that we live in a world where we want to create our own world. We want to be in charge of it. And I think this is going to help us. So we won’t have to accept that you die of cancer at a very young age.	Uhm, the influence of synthetic biology on society? I think it should be limited. If you look at for example at the story about braces with children. I think ten years ago one in ten children had a brace because it was really necessary. But at the moment every child has a brace. And we are going toward some kind of perfectionism. Everybody has to look perfect or as perfect as possible. And I think we don’t know what is perfect . . .	(I think) The influence of synthetic biology on the future society? I think there is a bright future with lots of possibilities. And lots of innovative solutions for things like . . . I just read an article about some scientist who does research on a plant . . . that creates its own resistance to bacteria. And that is great! I think it is really cool that these things are possible.	The influence of synthetic biology on the society is, I think, dependent on the context at that moment. It is not only a matter of how are we influence as society by the synthetic biology? But also how do we influence the synthetic biology field? It is a two-way street. And depending on the politics at that moment, the economy, war threats or not, tensions among citizens, how do we look at power of big companies—multinationals . . . Depending on these things we will give synthetic biology a place I think in our society.

**Table:**

Subtopic 2: What is the relationship between technology and human beings?	Well human beings as I know them, are curious. Curious about things that can be explored. I think it is inevitable that human beings keep on searching and exploring the world. I am a scientist so I know that if you are a scientist you cannot always oversee the consequences of what you are researching simply because you don’t know what it is going to be sometimes. So you will always keep on searching, I think we humans will always keep on searching. And it is possible that devices will be used in immoral ways or to hurt other beings. I think as a person we people, we are in control over technology, and we do not always choose what we find out, but we can choose what we do with it.	The world of technology and the human world. I think, of course, it influences each other. But I think we should be careful with that. If you see children in my school for example. They call them the Head Down generation. Everybody has his own screen with him or her. We could not foresee five years ago that it was going to be like this. And so fast it went. So I think it should be two separate worlds, which influence each other. That is my opinion.	The relationship between human beings and technology? I think they are one. They are all together, it is like one world. Humans create technology and technology develops. Did you see the movie HER? It is about a writer fall(ing) in love with an operating system. The operating system is self-learning. And they really start having are relationship together. So it is all one, technology and the real life. And their love, and . . . But in the end I think humans dominate the technology. You can reprogram the technology so things are changing.	The relationship between human being and technology. It is actually more and more becoming one. It is becoming one world, more and more a part of being a human, for which we logically start to internalize technological applications. What you often see is that people with new technological developments first are very distant and afraid. For example, if we jump back in the time to the moment at which the first trains and train tracks were being built. A lot of people—farmers for example—were really scared. When the trains would start driving, cows would get confused—like no more milk production. And humans thought it would be unhealthy to drive that much faster than humans naturally move themselves. At a certain moment people started trying it and discovered it delivered them benefits. Time and more efficiency. And then it became a part of their daily behavior. Then people do not even think consciously about it anymore. Then it (the technology) becomes part of you being a human.

**Table:**

Subtopic 3a: What is the adequate ethical approach to synthetic biology?	I can imagine that we will encounter ethical dilemmas in this field. I think you should always base your decisions on facts and not on fears. It is easy to be controlled by fears. But it is important to stick to the facts. So I, if it were me, I would choose someone who is capable and analyse if there is a real ethical problem and then make a decision. And not be guided by fear. I was thinking about nuclear energy. Because that is also, there is a lot of uproar about it and fear. And I know someone who is a nuclear physicist and knows a lot about it. And I always thought why can’t someone like him decide whether we are going to buy or going to place another nuclear reactor to produce energy? Why can’t it be someone like him and not politicians who do not know anything about it and are guided by fear?	Uhm ethics, I thought you would be coming to this. I think we have a lot of arrogance in science. There is a lot of research, and we think we know a lot about the world. But if you look at it exactly, every answer we give to a question gives another ten questions. So what do we really know about the consequences of what we are doing now? If you look for example, at the gender of a child that is conceived. Think about it if we could decide what gender it would be and you have a mother with four boys and she really wants a girl. And the girl is born. What kind of treatment is this girl getting from her parents? Because she is so much wanted. And how are they treating the other four boys? I don’t know. I can’t oversee, I can’t foresee what consequences there are. So well I think we have to be critical and look at the limits.	The ethical approach to synthetic biology? I think we need freedom. No rules. No structures, but just freedom. If you wanna be creative and think in new solutions, you don’t need so many rules and just. You just need freedom. More the “yes and” attitude, instead of “yes but.” And I think we should believe in the integrity of people who work with that. And they will find good ways to do so.	The adequate ethical approach for synthetic biology is mostly that we as society are going to carry the responsibility all together. It is needed that we converse with one another about it. That we engage in a dialogue with one another, in which we search for what unifies us. What is our mutual interest? What do we want? And how can we achieve that in such a way that everybody can agree with that? It is really important that we listen to each other carefully and in a respectful manner. That we do not judge too quickly but also give it time to develop new ethics for it: a vision.

**Table:**

Subtopic 3b: What is the role of citizens in the further development of synthetic biology?	The role of the citizen in the field of synthetic biology? I think citizens should be well informed so that they won’t be afraid of it. I think the decision-making should be with the experts. So the citizens should not be part of the debate about that. And if I look at myself, I am a physicist so I am not a biologist. I don’t know all the ins and outs of this. And I trust that the experts will have a good opinion about this. But I don’t have to intervene. I can leave it safely with the experts.	I think it’s, I should stay critical. That is what I am asking from myself. And I think the public should do as well. In this kind of developments. Maybe send letters to newspapers. Maybe on public media. You can post critical articles. You can ask people to stay, keep on thinking about these things. Of course there have to be laws from the government, but I am a bit insecure if that is really going to help. So I think everybody has his own responsibility to stay critical.	The public involvement? I think everybody should be able to contribute and to discuss. And everybody should TAKE their own influence in that. If you want to have influence in the development you should be heard and take the influence. For example when I started making music, I used the technology that was available. And things were developing and I just go with that flow. I was thinking, I did, I studied biology long time ago. And I was thinking perhaps I should also create some things with or experiment with synthetic biology. So perhaps I am going to do, make a kind of lab in my studio as well. And create something over here?!	I think that the role of the citizen is incredibly important. And everyone should feel that and take that responsibility. Actually in the way a democracy can only function when everyone participates and votes. In that way only can science and applications of it flourish; when people express themselves about it. And within that people should be willing to be critical, dive into it. Look at it from different perspectives, develop an opinion, exchange it, dare to change your initial assumptions and form new opinions. Stay in contact, keep moving, keep conversing. That is really important I think.

**Table:**

				What I will do, I will am planning to gather knowledge about it, develop a preliminary opinion based on that, and publish that. I have a blog and always invite people to respond. And I find it really interesting if people come back with well-elaborated arguments and opinions about it, which give me new ideas. Then I respond to it again, and I like open discussions, open conversations, and also the willingness to let go your initial thoughts. So what I would like to contribute in that is facilitating conversations; participating in forums. Or maybe even start new forums. That actually . . . Yes.

## Notes

1. Figures 1 and 2 both comprise quadrants. However, they map different understandings and cannot be transposed on top of each other.

## References

[bibr1-1075547017730585] Abelson J. Forest P. G. Eyles J. Smith P. Martin E. Gauvin F. P. (2003). Deliberations about deliberative methods: Issues in the design and evaluation of public participation processes. Social Science & Medicine, 57, 239-251. doi:10.1016/S0277-9536(02)00343-X 12765705

[bibr2-1075547017730585] Ancillotti M. Rerimassie V. Seitz S. B. Steurer W. (2016). An update of public perceptions of synthetic biology: Still undecided? NanoEthics, 10, 309-325. doi:10.1007/s11569-016-0256-3

[bibr3-1075547017730585] Avraamidou L. Osborne J. (2009). The role of narrative in communicating science. International Journal of Science Education, 31, 1683-1707. doi:10.1080/09500690802380695

[bibr4-1075547017730585] Balmer A. Martin P. (2008). Synthetic biology, social and ethical challenges (An independent review commissioned by the Biotechnology and Biological Sciences Research Council). Retrieved from http://www.bbsrc.ac.uk/documents/synthetic-biology-pdf/

[bibr5-1075547017730585] Bandelli A. Konijn E. (2011). An experimental approach to strengthen the role of science centers in the governance of science. In Marstine J. C. (Ed.), The Routledge companion to museum ethics (pp. 164-173). London, England: Routledge.

[bibr6-1075547017730585] Bandelli A. Konijn E. (2015). Public participation and scientific citizenship in the science museum in London: Visitors’ perceptions of the museum as a broker. Visitor Studies, 18, 131-149. doi:10.1080/10645578.2015.1079089

[bibr7-1075547017730585] Betten A. W. Broerse J. E. W. Kupper F. (2017). Dynamics of problem setting and framing in citizen discussions on synthetic biology. Public Understanding of Science. Advance online publication. doi:10.1177/0963662517712207 PMC5843019 28597721

[bibr8-1075547017730585] Boerwinkel D. J. Swierstra T. Waarlo A. J. (2014). Reframing and articulating socio-scientific classroom discourses on genetic testing from an STS perspective. Science & Education, 23, 485-507. doi:10.1007/s11191-012-9528-7

[bibr9-1075547017730585] Boldt J. (2016). Swiss watches, genetic machines, and ethics. An introduction to synthetic biology’s conceptual and ethical challenges. In Boldt J. (Ed.), Synthetic biology metaphors, worldviews, ethics, and law (pp. 1-12). Wiesbaden, Germany: Springer Fachmedien.

[bibr10-1075547017730585] Braun V. Clarke V. (2006). Using thematic analysis in psychology. Qualitative Research in Psychology, 3, 77-101. doi:10.1191/1478088706qp063oa

[bibr11-1075547017730585] Broerse J. E. W. De Cock Buning T. J. (2011). Public engagement in science and technology. In Chadwick R. (Eds.), Encyclopedia of applied ethics (pp. 674-684). London, England: Elsevier.

[bibr12-1075547017730585] Bucchi M. (2008). Chapter 5: Of deficits, deviations and dialogues. Theories on public communication of science. In Bucchi M. Trench B. (Eds.), Handbook of public communication of science and technology (pp. 57-73). London, England: Taylor & Francis.

[bibr13-1075547017730585] Carpini M. X. D. Cook F. L. Jacobs L. R. (2004). Public deliberations, discursive participation and citizen engagement: A review of the empirical literature. Annual Review of Political Science, 7, 315-344. doi:10.1146/annurev.polisci.7.121003.091630

[bibr14-1075547017730585] Davies S. McCallie E. Simonsson E. Lehr J. L. Duensing S. (2009). Discussing dialogue: Perspectives on the value of science dialogue events that do not inform policy. Public Understanding of Science, 18, 338-353. doi:10.1177/0963662507079760

[bibr15-1075547017730585] DeCuir-Gunby J. T. Marshall P. L. McCulloch A. W. (2011). Developing and using a codebook for the analysis of interview data: An example from a professional development research project. Field Methods, 23, 136-155. doi:10.1177/1525822X10388468

[bibr16-1075547017730585] Dewulf A. Bouwen R. (2012). Issue framing in conversations for change: Discursive interaction strategies for “doing differences.” Journal of Applied Behavioral Science, 48, 168-193. doi:10.1177/0021886312438858

[bibr17-1075547017730585] Drake L. E. Donohue W. A. (1996). Communicative framing theory in conflict resolution. Communication Research, 23, 297-322. doi:10.1177/009365096023003003.

[bibr18-1075547017730585] Grin J. van der Graaf H. (1996). Implementation as communicative action: An interpretive understanding of interaction between policy actors and target groups. Policy Sciences, 29, 291-319.

[bibr19-1075547017730585] Horst M. Michael M. (2011). On the shoulders of idiots: Re-thinking Science Communication as “event.” Science as Culture, 20(12), 283-306. doi:10.1080/09505431.2010.524199

[bibr20-1075547017730585] Knippels M.-C. Severiens S. E. Klop T. (2009). Education through fiction: Acquiring opinion-forming skills in the context of genomics. International Journal of Science Education, 31, 2057-2083.

[bibr21-1075547017730585] Korthals M. (2011). Deliberations on the life sciences: Pitfalls, challenges and solutions. Journal of Public Deliberation, 7, 8.

[bibr22-1075547017730585] Kupper F. Krijgsman L. Bout H. J. De Cock Buning T. (2007). The value lab: Exploring moral frameworks in the deliberation of values in the animal biotechnology debate. Science and Public Policy, 34, 657-670. doi:10.3152/030234207X264944

[bibr23-1075547017730585] Lambert S. D. Loiselle C. G. (2008). Combining individual interviews and focus groups to enhance data richness. Journal of Advanced Nursing, 62, 228-237. doi:10.1111/j.1365-2648.2007.04559.x 18394035

[bibr24-1075547017730585] Loeber A. Griessler E. Versteeg W. (2011). Stop looking up the ladder: Analyzing the impact of participatory technology assessment from a process perspective. Science and Public Policy, 38, 599-608.

[bibr25-1075547017730585] Lucivero F. (2016). Chapter 7: Scenarios as “Grounded explorations.” Designing tools for discussing the desirability of emerging technologies. In Lucivero F. (Ed.), Ethical assessments of emerging technologies (pp. 155-190). New York, NY: Springer. doi:10.1007/978-3-319-23282-9

[bibr26-1075547017730585] McKee M. (2003). Excavating our frames of mind: The key to dialogue and collaboration. Social Work, 48, 401-408. doi:10.1093/sw/48.3.401 12899287

[bibr27-1075547017730585] Müller O. (2016). Synthetic biology: On epistemological black boxes, human self-assurance, and the hybridity of practices and values. In Boldt J. (Ed.), Synthetic biology metaphors, worldviews, ethics, and law (pp. 31-46). Wiesbaden, Germany: Springer Fachmedien.

[bibr28-1075547017730585] Murmann M. Avraamidou L. (2014). Narrative as a learning tool in science centers: Potentials, possibilities and merits. Journal of Science Communication, 13(2), A02.

[bibr29-1075547017730585] Nisbet M. Markowitz E. M. (2014). Understanding public opinion in debates over biomedical research: Looking beyond political partisanship to focus on beliefs about science and society. PLoS One, 9, e88473. doi:10.1371/journal.pone.0088473 24558393 PMC3928253

[bibr30-1075547017730585] Owen R. Macnaghten P. M. Stilgoe J. (2012). Responsible Research and Innovation: From science in society to science for society, with society. Science and Public Policy, 39, 751-760. doi:10.1093/scipol/scs093

[bibr31-1075547017730585] Pineda M. V. Bernhardsson L. (2011). The great learning experience project: An attempt to understanding learning from the views of the millennial learners: Part 1. Retrieved from http://hv.diva-portal.org/smash/get/diva2:650706/FULLTEXT01.pdf

[bibr32-1075547017730585] Rogers E. M. (1962). Diffusion of innovations. New York, NY: Free Press.

[bibr33-1075547017730585] Rowe G. Frewer L. J. (2000). Public participation methods: A framework for evaluation. Science, Technology, & Human Values, 25, 3-29.

[bibr34-1075547017730585] Rowe G. Frewer L. J. (2005). A typology of public engagement mechanisms. Science, Technology, & Human Values, 29, 512-556. doi:10.1177/0162243904271724

[bibr35-1075547017730585] Schmidt M. Ganguli-Mitra A. Torgersen H. Kelle A. Deplazes A. Biller-Andorno N. (2009). A priority paper for the societal and ethical aspects of synthetic biology. Systems and Synthetic Biology, 3(1-4), 3-7. doi:10.1007/s11693-009-9034-7 19816794 PMC2759426

[bibr36-1075547017730585] Schmidt M. Meyer A. Cserer A. (2015). The Bio: Fiction film festival: Sensing how a debate about synthetic biology might evolve. Public Understanding of Science, 24, 619-635. doi:10.1177/0963662513503772 24164747 PMC4466099

[bibr37-1075547017730585] Skydsgaard M. A. Møller Andersen H. King H. (2016). Designing museum exhibits that facilitate visitor reflection and discussion. Museum Management and Curatorship, 31, 48-68. doi:10.1080/09647775.2015.1117237

[bibr38-1075547017730585] South J. B. Gabbitas B. Merrill P. F. (2008). Designing video-narratives to contextualize content for ESL learners: A design process case study. Interactive Learning Environments, 16, 231-243. doi:10.1080/10494820802114044

[bibr39-1075547017730585] Stilgoe J. Lock S. J. Wilsdon J. (2014). Why should we promote public engagement with science? Public Understanding of Science, 23, 4-15. doi:10.1177/0963662513518154 24434705 PMC5753839

[bibr40-1075547017730585] Stilgoe J. Owen R. Macnaghten P. (2013). Developing a framework for responsible innovation. Research Policy, 42, 1568-1580. doi:10.1016/j.respol.2013.05.008

[bibr41-1075547017730585] Sykes K. Macnaghten P. (2013). Responsible innovation: Opening up dialogue and debate. In Owen R. Bessant J. Heintz M. (Eds.), Responsible innovation: Managing the responsible emergence of science and innovation in society (pp. 85-108). Chichester, England: Wiley. doi:10.1002/9781118551424.ch5

[bibr42-1075547017730585] Torgersen H. Schmidt M. (2013). Frames and comparators: How might a debate on synthetic biology evolve? Futures, 48, 44-54. doi:10.1016/j.futures.2013.02.002 23805003 PMC3688360

[bibr43-1075547017730585] van der Meij M. G. Kupper F. Beers P. J. Broerse J. E. W (2016). Hybrid e-learning tool TransLearning: Video storytelling to foster vicarious learning within multi-stakeholder collaboration networks. International Journal of Lifelong Education, 35, 413-429. doi:10.1080/02601370.2016.1197331

[bibr44-1075547017730585] Van Kesteren B. J (1993). Applications of De Groot’s “learner report”: A tool to identify educational objectives and learning experiences. Studies in Educational Evaluation, 19, 65-86. doi:10.1016/S0191-491X(05)80057-4

[bibr45-1075547017730585] Verbeek P. P. C. C (2005). Techniek en de grens van de mens: De menselijke conditie in een technologische cultuur [Technology and the boundary of human beings: The human condition in a technological culture]. Wijsgerig Perspectief op Maatschappij en Wetenschap, 45(3), 6-17.

[bibr46-1075547017730585] Von Schomberg R (2013). A vision of Responsible Research and Innovation. In Owen R. Bessant J. Heintz M. (Eds.), Responsible innovation: Managing the responsible emergence of science and innovation in society (pp. 51-74). Chichester, England: Wiley.

[bibr47-1075547017730585] Walker C. A. Newcomb P. Cagle C. (2005). Age and ageism: Inhabiting the lives of healthy older adults through video-narratives. Journal of Nursing Education, 44, 283-285. 16021807 10.3928/01484834-20050601-09

